# The Clinicopathological Features and Genetic Alterations in Epstein–Barr Virus-Associated Gastric Cancer Patients after Curative Surgery

**DOI:** 10.3390/cancers12061517

**Published:** 2020-06-10

**Authors:** Wen-Liang Fang, Ming-Huang Chen, Kuo-Hung Huang, Chien-Hsing Lin, Yee Chao, Su-Shun Lo, Anna Fen-Yau Li, Chew-Wun Wu, Yi-Ming Shyr

**Affiliations:** 1 Division of General Surgery, Department of Surgery, Taipei Veterans General Hospital, Taipei 11217, Taiwan; khhuang@vghtpe.gov.tw (K.-H.H.); chewwunwu@gmail.com (C.-W.W.); ymshyr@vghtpe.gov.tw (Y.-M.S.); 2 School of Medicine, National Yang-Ming University, Taipei 11221, Taiwan; mhchen9@vghtpe.gov.tw (M.-H.C.); ychao@vghtpe.gov.tw (Y.C.); sslo@ymuh.ym.edu.tw (S.-S.L.); fyli@vghtpe.gov.tw (A.F.-Y.L.); 3 Center of Immuno-Oncology, Department of Oncology, Taipei Veterans General Hospital, Taipei 11217, Taiwan; 4 Genome Research Center, National Yang-Ming University, Taipei 11221, Taiwan; jameslin@fcbiotech.com.tw; 5 Department of Surgery, National Yang-Ming University Hospital, Yilan 26058, Taiwan; 6 Department of Pathology, Taipei Veterans General Hospital, Taipei 11217, Taiwan

**Keywords:** Epstein–Barr virus, intestinal/solid, poorly cohesive, lymphoepithelioma-like, PD-L1, immunotherapy, targeted therapy

## Abstract

Background: Epstein–Barr virus (EBV)-associated gastric cancer (GC) is one of four major gastric cancer types and is traditionally considered to be related to lymphoepithelioma-like GC. Few studies have investigated the clinical significance of EBV infection in intestinal/solid type, diffuse (poorly cohesive) type, and lymphoepithelioma-like GC. Methods: A total of 460 GC patients receiving curative surgery were enrolled. The clinicopathological features, genetic alterations and prognoses were compared between patients with and without EBV infection. Results: EBV-positive GC patients (*n* = 43) had more tumors located in the upper and middle stomach, more common in lymphoepithelioma-like carcinoma, more lymphoid stroma, fewer Helicobacter pylori infections, and higher programmed death-ligand 1 (PD-L1) expression than EBV-negative GC patients. For intestinal/solid type GC, EBV-positive tumors were more likely to be located in the upper and middle stomach, have more lymphoid stroma, fewer Helicobacter pylori infections, higher PD-L1 expression, and more liver metastases than EBV-negative tumors. For diffuse (poorly cohesive) type GC, EBV-positive tumors were more likely to be located in the upper stomach, and have more lymphoid stroma than EBV-negative tumors. For lymphoepithelioma-like GC, EBV-positive tumors had more PI3K/AKT pathway mutations than EBV-negative tumors. Conclusions: Intestinal/solid type GC patients with EBV-positive tumors were associated with higher PD-L1 expression and more liver metastases, while lymphoepithelioma-like GC patients with EBV-positive tumors had more PI3K/AKT pathway mutations. Immunotherapy and targeted therapy may be beneficial for these groups of patients. Routine EBV survey is recommended in GC.

## 1. Introduction

Epstein–Barr virus (EBV)-associated gastric cancer (GC) is one of the four major types of GC [1]. Compared with EBV-negative GC, EBV-positive GC is associated with lymphocyte infiltration, genetic mutations in PIK3CA and ARID1A and the hypermethylation of CpG islands [1,2].

EBV-encoded small RNAs (EBERs) are abundantly expressed in nearly all the neoplastic cells of the tumor tissue, and the EBER-in situ hybridization (ISH) technique is served as a gold standard to define EBV-associated GC [3]. EBV-positive GC which is known to have high programmed death-ligand 1 (PD-L1) expression mainly has morphologic features of GC with lymphoid stroma, that is, so-called lymphoepithelioma-like GC, with similarities to nasopharyngeal carcinoma and different from the typical intestinal-type or diffuse-type GC [4].

The correlation between EBV infection and patient prognosis in GC is controversial; some studies reported a good prognosis in EBV-positive GC [4,5,6], while some reported a poor prognosis [7,8]. A high viral load of EBV may contribute to PD-L1 expression and is associated with cancer progression and poor prognosis in GC [7]. EBV infection was reported to be associated with a poor prognosis in intestinal-type GC and a better prognosis in diffuse-type GC; however, EBV infection was a prognostic factor only in diffuse-type GC [9]. To date, the prognostic value of EBV infection and its correlation with PD-L1 expression in intestinal-type and diffuse-type GC is still obscure.

According to the new WHO classification for histological classifications of GC [10], intestinal-type GC includes papillary GC and well-differentiated and moderate-differentiated tubular GC; solid-type GC is defined as poorly differentiated tubular GC; diffuse-type GC is defined as poorly cohesive type GC. In the present study, we separated all the enrolled GC patients into three groups: Intestinal/solid type GC, diffuse (poorly cohesive) type GC, and lymphoepithelioma-like GC. The aim of the present study was to investigate the clinicopathological features and genetic alterations between EBV-positive and EBV-negative GC patients. Moreover, we analyzed the clinical significance and incidence of EBV infection in aforementioned three groups of GC patients.

## 2. Results

### 2.1. Clinicopathologic Characteristics

Among the 460 patients, 43 (9.3%) had EBV-positive GC. Patients with EBV-positive GC were associated with having more tumors located in the upper and middle stomach, more common in lymphoepithelioma-like GC, more lymphoid stroma, fewer Helicobacter pylori (HP) infections, higher PD-L1 expression, and more PI3K/AKT pathway mutations than patients with EBV-negative GC (Table 1).

**Figure 1:**
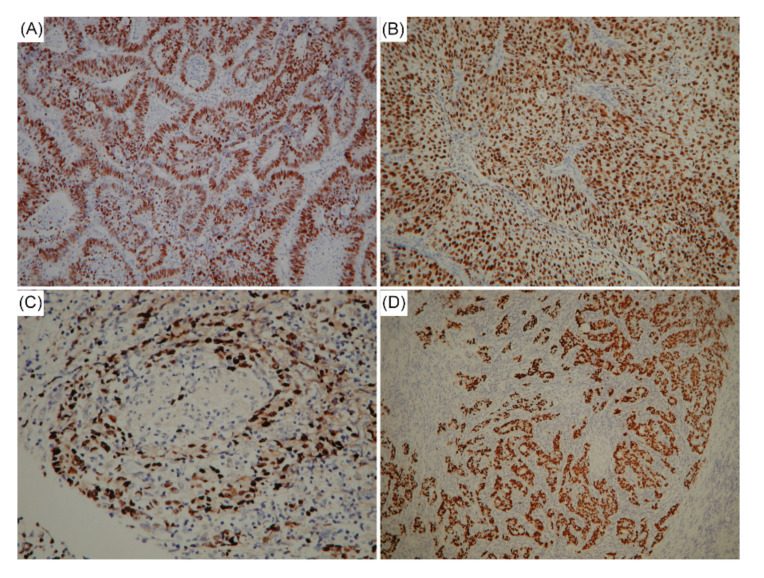
Positive EBV-encoded small RNA in situ hybridization (EBER ISH) results in each histological type of GC are shown as follows: (**A**) Intestinal type GC; (**B**) solid type GC; (**C**) diffuse (poorly cohesive) type GC; and (**D**) lymphoepithelioma-like GC.

Positive EBER IHC staining results are shown in Figure 1. The frequency of EBV-positive tumors was the highest in the lymphoepithelioma-like GC (36.7% (11/30)), followed by intestinal/solid type (7.8% (17/218)) and diffuse (poorly cohesive) type GC (7.1% (15/212)). The results demonstrated that the histology of EBV-positive GC is not limited for lymphoepithelial carcinoma; EBV-positive GC can appear in intestinal/solid type GC and diffuse (poorly cohesive) type GC.

**Figure 2:**
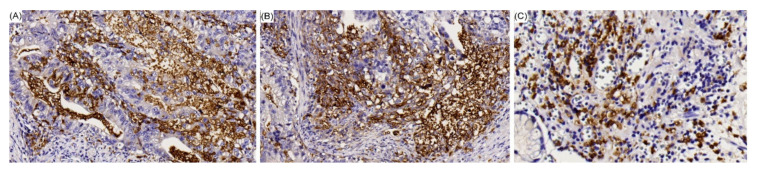
Positive PD-L1 IHC staining results in each histological type of GC are shown as follows: (**A**) Intestinal type GC; (**B**) diffuse (poorly cohesive) type GC; and (**C**) lymphoepithelioma-like GC.

For intestinal/solid type GC (Table 2), patients with EBV-positive GC had more tumors located in the upper and middle stomach, more lymphoid stroma, fewer HP infections, and higher PD-L1 expression (Figure 2) than those with EBV-negative GC.

For diffuse (poorly cohesive) type GC (Table 2), patients with EBV-positive GC had more tumors located in the upper stomach and more lymphoid stroma than those with EBV-negative GC.

For lymphoepithelioma-like GC (Table 2), patients with EBV-positive GC had more PI3K/AKT pathway mutations than EBV-negative GC.

### 2.2. Initial Recurrence Patterns

The median follow-up period was 57.8 months. There was no significant difference in the initial recurrence pattern between EBV-positive and EBV-negative GC patients.

As shown in Table 3, for patients with intestinal/solid type GC, those with EBV-positive tumors were more likely to experience distant metastases than intestinal/solid type GC patients without EBV infection (52.9% vs. 27.9%, *p* = 0.030), especially liver metastases (35.3% vs. 11.9%, *p* = 0.001). For diffuse (poorly cohesive) type GC or lymphoepithelioma-like GC, there was no difference in the initial recurrence pattern between patients with EBV-positive and EBV-negative GC.

### 2.3. Survival Analysis

**Figure 3:**
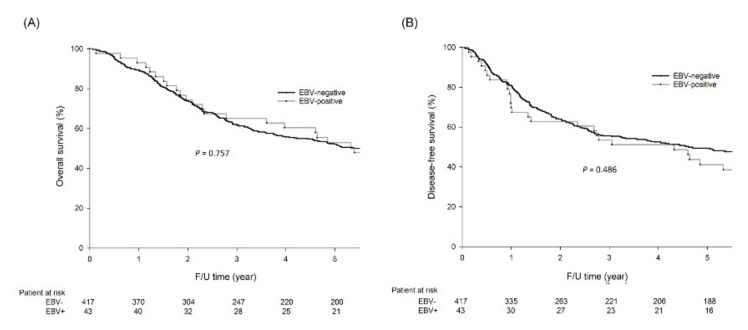
The five-year overall survival (OS) rates (52.9% vs. 52.2%, *p* = 0.757) and disease-free survival (DFS) rates (41.1% vs. 49.5%, *p* = 0.486) were not significantly different between EBV-positive and EBV-negative GC patients. The OS and DFS curves are shown as follows: (**A**) OS curves of all GC patients; (**B**) DFS curves of all GC patients.

For all the patients enrolled, the 5-year overall survival (OS) rates (52.9% vs. 52.2%, *p* = 0.757, Figure 3A) and disease-free survival (DFS) rates (41.1% vs. 49.5%, *p* = 0.486, Figure 3B) were not significantly different between EBV-positive and EBV-negative GC.

For patients with intestinal/solid type GC, there was no difference in OS (64.7% vs. 55.9%, *p* = 0.664) and DFS (46.3% vs. 52.5%, *p* = 0.970) rates between those with EBV-positive and EBV-negative GC. For patients with diffuse (poorly cohesive) type GC, no difference in OS (51.9% vs. 48.9%, *p* = 0.741) and DFS (45.0% vs. 46.5%, *p* = 0.720) was observed between those with EBV-positive and EBV-negative GC. For patients with lymphoepithelioma-like GC, no difference in OS (36.4% vs. 47.4%, *p* = 0.215) and DFS (27.3% vs. 47.4%, *p* = 0.124) was observed between those with EBV-positive and EBV-negative GC.

As shown in Table 4 and Table 5, the univariate analysis demonstrated that age, gender, tumor size, and pathological TNM stage were associated with OS and DFS. The aforementioned four variables were included in a multivariate Cox proportional hazards model to adjust for the effects of covariates. The multivariate analysis demonstrated that age, tumor size, and pathological TNM stage were independent prognostic factors affecting OS and DFS (Table 4 and Table 5).

## 3. Discussion

Our results showed that EBV-positive GC patients were more likely to have PD-L1 expression and similar OS and DFS rates compared with EBV-negative GC patients. For intestinal/solid type GC patients, EBV-positive tumors were associated with greater PD-L1 expression and more liver metastases than EBV-negative tumors; for lymphoepithelioma-like GC patients, EBV-positive tumors were associated with more PI3K/AKT pathway mutations than EBV-negative tumors.

In 1990, EBV was first detected in lymphoepithelial carcinoma of the stomach, which was similar to undifferentiated nasopharyngeal lymphoepithelioma [11]. However, EBV was found not only in the rare gastric lymphoepithelial carcinoma but also in gastric adenocarcinomas [12,13], including intestinal-type GC and diffuse-type GC [14], which was confirmed by polymerase chain reaction (PCR) and ISH in a variety of studies [15,16,17]. EBV-positive status can serve as a biomarker for immunotherapy in GC patients [18], which might be due to a higher level of PD-L1 [19] and more tumor infiltrating lymphocytes in EBV-associated GC [20]. Previous studies demonstrated that EBV-associated GC was more common in diffuse-type than in intestinal-type GC (9.9 vs. 6.5%) [21]. Our results showed that the frequency of EBV-positive tumors was the highest in lymphoepithelioma-like GC (36.7%), followed by intestinal/solid type GC (7.8%) and diffuse (poorly cohesive) type GC (7.1%). Consequently, a proportion of EBV-associated GC patients may lose the opportunity to receive immunotherapy if they are diagnosed based on lymphoepithelioma-like GC only. A routine examination for EBV infection in GC patients is recommended. Our results may have an impact on clinical practice for GC treatment in the future.

An interesting finding that the incidence of PI3K/AKT pathway mutation was 25.9% (11/43) in EBV-positive GC patients in the present study, which was similar to findings in a Korean study [22] (25%, 28/112) and significantly lower than that in The Cancer Genome Atlas (TCGA) database (80%, 21/26). The discrepancy might be due to racial differences and the relatively small patient number of EBV-positive tumors in all three studies. Gastric carcinogenesis is complicated and variable among individuals; consequently, more patients enrolled from different areas and races are required to validate our results. Our previous report demonstrated that GC patients with HP infection was associated with a lower frequency of PI3K/AKT pathway mutations than those without HP infection [23]. In the present study, the incidence of PI3K/AKT pathway mutations according to the status of EBV/HP infection is 16.7% in EBV+/HP+ tumors, 27.0% in EBV+/HP- tumors, 9.3% in EBV-/HP+ tumors, and 16.9% in EBV-/HP- tumors. It seems that the incidence of PI3K/AKT pathway mutations is the highest in GC patients with EBV infection only, and the lowest in GC patients with HP infection only. We hypothesize that the incidence of PI3K/AKT pathway mutations in EBV-associated GC patients may be influenced by the coexistence of HP infection, which might explain the discrepancy between studies endemic for HP. It was reported that the PI3K/AKT pathway played an important role in EBV-associated cancer [24,25,26]. The activation of the PI3K/AKT pathway can result in metastasis and drug resistance to chemotherapy [27,28]. PI3K inhibitor, LY294002, has been shown to increase the effect of 5-fluorouracil in an EBV-associated GC cell line [29]. Our results demonstrated that PI3K/AKT pathway mutations were more common in EBV-associated GC patients with a frequency of 25.9%; consequently, targeted therapy may be beneficial for this subgroup of patients. Further in vitro and in vivo studies regarding the relationship among EBV, HP, and the PI3K/AKT pathway in GC is required to validate our hypothesis.

Our results showed that PD-L1 expression was significantly higher in EBV-positive GC patients than in those who had EBV-negative GC, which was similar to the findings in previous reports [20]. As a result, immunotherapy may be applicable for EBV-positive GC patients. It was reported that PD-L1 expression was associated with poorly differentiated, intestinal-type, and high lymphoid response GC [30]. However, some studies demonstrated that in EBV-positive GC, PD-L1 expression was more common in diffuse-type than in intestinal-type GC [20]. In the present study, EBV-positive GC was associated with significantly more PD-L1 expression than EBV-negative GC, especially in intestinal/solid type GC. However, in diffuse (poorly cohesive) type GC, the PD-L1 expression was not significantly different between EBV-positive and EBV-negative GC. It is interesting regarding the discrepancy of results between the present study and others [20]. In the present study, patients with EBV infection were associated with fewer HP infections than in those without EBV infection. As we know, HP infection is associated with the development of GC, and it plays an important role in the carcinogenesis of both intestinal-type and diffuse-type GC. Furthermore, HP infection was reported to be associated with PD-L1 expression [30]. As a result, HP infection might affect the PD-L1 expression in intestinal-type and diffuse-type GC. The discrepancy of PD-L1 expression in different histological types of GC with or without EBV infection might be influenced by the coexistence of HP infection. More patients enrolled from different countries and races are required to validate our results and hypotheses.

The correlation between EBV infection and patient prognosis is controversial; some studies reported a better prognosis [4,5,6], and others reported a worse prognosis [7,8]. It was reported that compared with EBV-negative GC, EBV-positive GC was associated with a worse survival in patients with intestinal-type tumors and a better survival in patients with diffuse-type tumors; moreover, EBV infection was an independent prognostic factor only in patients with diffuse-type GC [8]. In the present study, EBV infection was not associated with patient prognosis in either intestinal/solid type, diffuse (poorly cohesive) type, or lymphoepithelioma-like GC patients. The discrepancy might be due to small sample size, different races, environmental factors, etc. Enrollment of a larger sample or a meta-analysis are required to investigate the prognostic role of EBV infection in GC patients.

Our result demonstrated that in intestinal/solid type GC, EBV-positive patients had more PD-L1 expression and liver metastases than EBV-negative patients. Physicians should be aware of liver metastasis in this subgroup of patients during follow-up. Examination of PD-L1 expression is recommended for improving patient prognosis in the cases of tumor recurrence. Figure 2 demonstrated PD-L1 expression in different histological types of GC. Questions have been raised concerning the technical aspects of determining a positive or negative test for PD-L1. These included the specificity of several clones of anti-human PD-L1 antibodies for IHC staining and the artifacts that may be derived from different techniques for tissue fixation and antigen retrieval, especially when distant tumor niche sites are involved [31,32]. In this study, the definition of positive expression of PD-L1 is a combined positive score (CPS) of >1. In the recent KEYNOTE 059 study, anti-PD-L1 antibody, pembrolizumab, demonstrated promising activity in patients with advanced GC who had previously been treated with least a 2nd line therapy. Therefore, the Food and Drug Administration (FDA) grants accelerated approval of pembrolizumab for PD-L1-positive (CPS > 1) GC. Anti-PD-L1 antibody with 22C3 (Dako, Carpinteria, CA, USA) and CPS were adapted in the KEYNOTE 059 study. We adapted the same antibody and analytic platform in this study [33]. The caveat is that some patients who are tested positive for PD-L1 may not respond to immunotherapy, and more importantly, some patients who are tested negative may still respond, making it an imperfect biomarker. Another important biomarker for immune checkpoint blockades (ICB) is tumor microenvironment, so-called “hot” tumors. Hot tumors are characterized by the accumulation of proinflammatory cytokines and T cell infiltration and have a better response rate to ICB treatment. Indeed, EBV associated lymphoepithelial carcinoma is a typical hot tumor (Figure 1D), which can partially explain why EBV associated GC might be a good candidate for ICB [18].

The role of the tumor milieu in mediating cancer progression can affect the immune infiltrate in both solid and hematologic tumors. Indeed, the cytokine- and cell-adhesion-dependent bone marrow niche and stromal microenvironment support new vessel formation and cancer proliferation, irrespective of immune surveillance [34]. For instance, EBV appears to impact the immune milieu and the patient’s survival, being implicated in immune status equilibrium, a major driver of GC initiation [35]. This intimate interaction between GC cells, the microenvironment via bystanders, i.e., endothelial cells, and CD8+ T cells creates a permissive immune microenvironment that allows undisturbed cancer proliferation in both solid and hematological malignancies [36,37]. Indeed, immune dysfunction leads to infections that are the major cause of mortality, and there is evidence of fewer infections in subcutaneous immunoglobulins-treated patients. It has been shown that immunoglobulin is effective in the treatment of secondary conditions or other viral infections such as EBV [38,39].

To date, several clinical trials investigated the use of immune checkpoint inhibitors (ICI) in GC and other gastrointestinal tract tumors [40,41]. According to the TCGA database [1], EBV-associated GC displays recurrent PIK3CA mutations, extreme DNA hypermethylation, and PD-L1 amplification. This classification provides further insight into the possible application of personalized therapy with ICI targeting PD-L1. In the present study, none of the enrolled patients received immunotherapy, thus we could not analyze the treatment response to immunotherapy. According to the novel findings of the present study, our future study will focus on the treatment response to immunotherapy for EBV-associated tumors in intestinal/solid type GC, diffuse (poorly cohesive) type GC, and lymphoepithelioma-like GC. We believe that comprehensive genetic analysis for these subgroups of patients using next-generation sequencing and investigation of the correlation between genetic alterations and response to immunotherapy is required and may have clinical application.

It is interesting that EBV infection is either a causative or a regulatory factor for GC. A meta-analysis reported that EBV infection increased the risk of malignancy [30], which indicated that EBV infection might be a causative factor for GC. However, in addition to EBV infection, HP infection is also a risk factor for GC [42]. Downregulation of tumor-specific immune responses was more frequent in patients without HP infection than in patients with HP infection [43]. Patients with HP infection were associated with a better prognosis than patients without HP infection [23,44], which might be due to more tumor-specific immune responses in patients with HP infection. A synergistic relationship between EBV and HP infection has been reported [45]. During the infection with both EBV and HP, immune responses are activated, which leads to gastric inflammation, gastric carcinogenesis, and proliferation of both HP and EBV. Immortalization of EBV and HP results in a high concentration of anti-apoptotic Bcl-2 protein and an increase in gastric oncogenesis [45]. Furthermore, EBV and HP infections could reduce the number of tumor suppressor genes, which also leads to gastric carcinogenesis. Consequently, EBV infection might also be a regulatory factor in GC with HP infection.

There are some limitations in the present study. First, this is a retrospective study and bias could exist. Second, the number of patients with EBV infection is limited due to a relative low incidence of EBV infection in GC cases. Third, this is a single-center study, and the components and races of our patient group might not be as variable as in multi-center studies.

## 4. Materials and Methods

### 4.1. Patients and Sample Collection

Between 2005 and 2012, a total of 460 patients with GC who underwent curative resection and had tissue samples available in a single institute were enrolled in this study.

The samples were meticulously dissected, and the tumor tissues and corresponding normal mucosa tissues were collected. The pathological staging of GC was performed according to the 8th American Joint Committee on Cancer/Union for International Cancer Control (AJCC/UICC) tumor, node, metastasis (TNM) classification [46].

Before surgery, all patients underwent chest radiography, abdominal sonography, or an abdominal computed tomography (CT) scan for tumor staging. A total or distal subtotal gastrectomy was performed depending on the distance between the cardia and the tumor. A subtotal gastrectomy is the standard procedure for distal GC, whereas a total gastrectomy is the common procedure for proximal GC. Regarding the extent of the lymphadenectomy, a minimum of a D1 + dissection was performed for early GC, whereas at least a D2 dissection was performed for advanced GC. For the D2 dissections, a combined-organ resection was sometimes performed to achieve curative resection.

All samples were collected from the biobank of our hospital and all samples were already-existing collected and anonymized. All procedures followed were in accordance with the ethical standards of the responsible committee on human experimentation and with the Helsinki Declaration of 1964 and later versions. The ethics committees of Taipei Veterans General Hospital reviewed and approved this study (No. 2015-03-002A).

### 4.2. Follow-Up

Follow-up assessments were performed every 3 months for the first 3 years after surgery and every 6 months thereafter until the patient’s death. Follow-up procedures included medical history, physical examinations, routine blood tests, liver function tests, measurements of tumor markers (e.g., carcinoembryonic antigen and carbohydrate antigen 19-9), chest radiography, abdominal sonography, and abdominal CT scan. Tumor recurrence was diagnosed by biopsies or imaging studies when biopsies were not obtained. Patients with tumor recurrence were eligible to receive 5-fluorouracil (FU)-based chemotherapy. None of the patients enrolled received preoperative chemotherapy. Since 2008, adjuvant therapy (such as S-1) has been prescribed for stage II or stage III disease after curative surgery at our hospital due to its proven survival benefit [47].

### 4.3. Identification of HP Infection

Fresh frozen or paraffin-embedded gastric tissues were used, and both tumor tissue and nontumor tissues were tested for HP infection. The reference sequence of the HP reference genome (GenBank: AE000511.1) was used to design PCR forward (AAGCTTACTTTCTAACACTAACGC) and reverse (AAGCTTTTAGGGGTGTTAGGGGTTT) primers. PCR was used for the identification of HP infection, as described in a previous study [23].

### 4.4. EBV Detection

EBV infection was investigated using histological sections obtained from formalin-fixed paraffin-embedded (FFPE) tissue blocks. EBV was identified using the ISH technique for the detection of EBERs in FFPE tissue samples. According to the manufacturer’s instruction, the EBER ISH was performed using the EBV Probe/Antibody ISH Kit (Leica Biosystems Newcastle Ltd., Newcastle-upon-Tyne, UK) in association with Ultra Vision Large Volume Detection System Anti-Polyvalent, HRP (Thermo Fisher Scientific, Fremont, CA, USA) as used previously report [48]. Positive staining at the site of hybridization (nucleus) was interpreted as positive for EBV. Positive EBER ISH staining results in intestinal-type GC (Figure 1A), solid-type GC (Figure 1B), diffuse (poorly cohesive) type GC (Figure 1C), and lymphoepithelial carcinoma (Figure 1D) were shown.

### 4.5. Mutation Analysis of GC-Related Genes Based on MassARRAY

As described in previous studies [23,49], we used MassARRAY to detect mutations in nine GC-related genes, including TP53, ARID1A, PTEN, PIK3CA, AKT1, AKT2, AKT3, KRAS, and BRAF. Among these genes, PTEN, PIK3CA, AKT1, AKT2, and AKT3 were analyzed to identify mutations in the PI3K/AKT pathway genetic mutations.

### 4.6. Microsatellite Instability Analysis

As described in a previous study [50], five reference microsatellite markers, including D5S345, D2S123, D17S250, BAT25 and BAT26, were used to determine microsatellite instability (MSI). MSI-high (MSI-H) was defined as ≥2 loci of instability with 5 markers, while MSI-stable (MSS) was defined as one loci or without loci of MSI.

### 4.7. IHC Staining of PD-L1

IHC staining was performed using a PD-L1 IHC 22C3 pharmDx kit on a Dako ASL48 platform according to the manufacturer’s recommendations [51]. The CPS was calculated. The CPS consisted of the number of PD-L1-stained cells, including tumor cells, lymphocytes, and macrophages, relative to the number of all viable tumor cells. A CPS score of ≥1 was interpreted as positive PD-L1 expression.

### 4.8. Statistical Analysis

Statistical analyses were performed with IBM SPSS Statistics 25.0 (IBM Corp., Armonk, NY, USA). The categorical data were compared using the χ2 test with Yates’ correction or Fisher’s exact test. The overall survival (OS) was measured from the date of the operation to the date of death or the final follow-up. The disease-free survival (DFS) was defined as the length of time after GC surgery during which the patient survived without tumor recurrence. The distributions of OS and DFS were estimated using the Kaplan–Meier method. Cox proportional hazards models were used to explore the association of the clinical parameters with OS. A *p* value of < 0.05 was considered statistically significant.

## 5. Conclusions

The present study demonstrated that patients with EBV-positive intestinal/solid type GC had higher PD-L1 expression and more liver metastases than those with EBV-negative GC. Patients with EBV-positive lymphoepithelioma-like GC had more PI3K/AKT pathway mutations than those with EBV-negative GC. Immunotherapy and targeted therapy may be beneficial for these subtypes of GC. A routine analysis for EBV infection is recommended for GC patients.

## Figures and Tables

**Table 1:** Clinical profile in all gastric cancer (GC) patients with or without Epstein–Barr virus (EBV) infection.

Variables	EBV-Negative *n* = 417 *n* (%)	EBV-Positive *n* = 43 *n* (%)	*p* Value
Age (year)			0.794
<65	166 (39.8)	18 (41.9)	
≥65	251 (60.2)	25 (58.1)	
Gender (M/F)	294/123	35/8	0.132
Tumor size (<5/≥ 5 cm)	163/254	16/27	0.810
Tumor location			**<0.001**
Upper stomach	67 (16.1)	17 (39.5)	
Middle stomach	136 (32.6)	18 (41.9)	
Lower stomach	201 (48.2)	9 (18.6)	
Whole stomach	13 (3.1)	0	
Cell differentiation			0.540
Poor	225 (54.0)	26 (60.5)	
Moderate	185 (44.4)	17 (39.5)	
Well	7 (1.6)	0	
Histological type			**<0.001**
Intestinal/solid type	201 (48.2)	17 (39.5)	
Diffuse (poor cohesive) type	197 (47.2)	15 (34.9)	
Lymphoepithelioma-like	19 (4.6)	11 (25.6)	
Lymphovascular invasion	295 (70.7)	29 (67.4)	0.651
Lymphoid stroma	49 (11.8)	18 (41.9)	**<0.001**
MSI status			0.640
MSI-L/S	379 (90.9)	40 (93.0)	
MSI-H	38 (9.1)	3 (7.0)	
HP infection	151 (36.2)	6 (14.0)	**0.003**
*PIK3CA* amplification	191 (45.8)	15 (34.9)	0.117
*PD-L1* expression	120 (28.8)	20 (46.5)	**0.016**
Genetic mutation			
*PI3K/AKT* pathway	59 (14.1)	11 (25.9)	**0.047**
*ARID1A*	50 (12.0)	3 (7.0)	0.327
*TP53*	56 (13.4)	3 (7.0)	0.228
*KRAS*	10 (2.4)	0	0.305
*BRAF*	3 (0.7)	0	0.577
Pathological T category			0.995
T1/2/3/4	63/71/142/141	7/7/15/14	
Pathological N category			0.670
N0/1/2/3	134/68/106/109	14/10/9/10	
Pathological TNM Stage			0.996
I/II/III	87/119/211	9/12/22	

EBV: Epstein–Barr virus; HP: Helicobacter pylori; MSI: Microsatellite instability; MSI-H: Microsatellite instability-high; MSI-L/S: Microsatellite instability-low/stable; TNM: Tumor, Node; Metastasis. Bold: Statistically significant.

**Table 2:** Clinical profile in GC patients with or without EBV infection.

Variables	Intestinal/Solid Type GC			Diffuse (Poorly Cohesive) Type GC			Lymphoepithelioma-Like GC		
	EBV-Negative *n* = 201 *n* (%)	EBV-Positive *n* = 17 *n* (%)	*p* Value	EBV-Negative *n* = 197 *n* (%)	EBV-Positive *n* = 15 *n* (%)	*p* Value	EBV-Negative *n* = 19 *n* (%)	EBV-Positive *n* = 11 *n* (%)	*p* Value
Age (year)			0.671			0.818			0.643
<65	61 (30.3)	6 (35.3)		98 (49.7)	7 (46.7)		7 (36.8)	5 (45.5)	
≥65	140 (69.7)	11 (64.7)		99 (50.3)	8 (53.3)		12 (63.2)	6 (54.5)	
Gender (M/F)	163/38	15/2	0.465	116/81	12/3	0.107	15/4	8/3	0.698
Tumor size (<5/≥5 cm)	82/119	7/10	0.976	75/122	6/9	0.882	6/13	3/8	0.804
Tumor location			**0.019**			**0.001**			0.082
Upper stomach	38 (18.9)	6 (35.3)		25 (12.7)	7 (46.7)		4 (21.1)	4 (36.4)	
Middle stomach	55 (27.4)	7 (41.2)		77 (39.1)	6 (40.0)		4 (21.1)	5 (45.5)	
Lower stomach	106 (52.7)	4 (23.5)		84 (42.6)	2 (13.3)		11 (57.8)	2 (18.1)	
Whole stomach	2 (1.0)	0		11 (5.6)	0		0	0	
Cell differentiation			0.721			0.608			0.685
Poor	37 (18.4)	4 (23.5)		170 (86.3)	12 (80.0)		18 (94.7)	10 (90.9)	
Moderate	159 (79.1)	13 (76.5)		25 (12.7)	3 (20.0)		1 (5.3)	1 (9.1)	
Well	5 (2.5)	0		2 (1.0)	0		0	0	
Lymphovascular invasion	144 (71.6)	10 (58.8)	0.265	137 (69.5)	10 (66.4)	0.816	14 (73.7)	9 (81.8)	0.612
Lymphoid stroma	27 (13.4)	6 (35.3)	**0.016**	5 (2.5)	2 (13.3)	**0.024**	19 (100)	11 (100)	
MSI status			0.917			0.841			-
MSI-L/S	179 (89.1)	15 (88.2)		181 (91.9)	14 (93.3)		19 (100)	11 (100)	
MSI-H	22 (10.9)	2 (11.8)		16 (8.1)	1 (6.7)		0	0	
HP infection	59 (29.4)	0	**0.009**	88 (44.7)	3 (20.0)	0.063	4 (21.1)	3 (27.3)	0.698
*PIK3CA* amplification	80 (39.8)	3 (17.6)	0.071	103 (52.3)	7 (46.7)	0.675	8 (42.1)	5 (45.5)	0.858
*PD-L1* expression	51 (25.4)	9 (52.9)	**0.019**	62 (31.5)	5 (33.3)	0.881	7 (36.8)	6 (54.5)	0.346
Genetic mutation									
*PI3K/AKT* pathway	40 (19.9)	6 (35.3)	0.135	19 (9.6)	2 (13.3)	0.645	0	3 (27.3)	**0.016**
*ARID1A*	32 (15.9)	3 (17.6)	0.852	18 (9.1)	0	0.221	0	0	-
*TP53*	20 (10.0)	1 (5.9)	0.585	31 (15.7)	1 (6.7)	0.344	5 (26.3)	1 (9.1)	0.256
*KRAS*	9 (4.5)	0	0.373	1 (0.5)	0	0.782	0	0	-
*BRAF*	3 (1.5)	0	0.612	0	0	-	0	0	-
Pathological T category			0.311			0.554			0.636
T1/2/3/4	27/44/63/67	5/2/5/5		34/22/71/70	1/2/7/5		2/5/8/4	1/3/3/4	
Pathological N category			0.300			0.584			0.991
N0/1/2/3	75/36/54/36	8/4/1/4		56/26/47/68	4/3/5/3		3/6/5/5	2/3/3/3	
Pathological TNM Stage			0.474			0.717			0.610
I/II/III	47/66/88	6/4/7		37/46/114	2/4/9		3/7/9	1/4/6	

EBV: Epstein–Barr virus; HP: Helicobacter pylori; MSI: Microsatellite instability; MSI-H: Microsatellite instability-high; MSI-L/S: Microsatellite instability-low/stable; TNM: Tumor, Node, Metastasis; Bold: Statistically significant.

**Table 3:** The initial recurrence pattern in GC patients.

Initial Recurrence Pattern	Intestinal/Solid Type GC			Diffuse (Poorly Cohesive) Type GC			Lymphoepithelioma-Like GC		
	EBV-Negative *n* = 201 *n* (%)	EBV-Positive *n* = 17 *n* (%)	*p* Value	EBV-Negative *n* = 197 *n* (%)	EBV-Positive *n* = 15 *n* (%)	*p* Value	EBV-Negative *n* = 19 *n* (%)	EBV-Positive *n* = 11 *n* (%)	*p* Value
Total patients with recurrence	66 (32.8)	9 (52.9)	0.094	71 (36.0)	4 (26.7)	0.464	5 (26.3)	4 (36.4)	0.563
Locoregional recurrence	32 (15.9)	2 (11.8)	0.650	26 (13.2)	2 (13.3)	0.988	3 (15.8)	1 (9.1)	0.603
Distant metastasis	56 (27.9)	9 (52.9)	**0.030**	61 (31.0)	3 (20.0)	0.373	4 (21.1)	3 (27.3)	0.698
Peritoneal dissemination	21 (10.4)	2 (11.8)	0.865	35 (17.8)	2 (13.3)	0.663	2 (10.5)	1 (9.1)	0.900
Hematogenous metastasis	31 (15.4)	7 (41.2)	**0.007**	26 (13.2)	1 (6.7)	0.465	2 (10.5)	2 (18.2)	0.552
Liver	24 (11.9)	6 (35.3)	**0.001**	13 (6.6)	1 (6.7)	0.992	2 (10.5)	0	0.265
Lung	3 (1.5)	1 (5.9)	0.195	5 (2.5)	0	0.532	1 (5.3)	1 (9.1)	0.685
Bone	6 (3.0)	1 (5.9)	0.515	5 (2.5)	0	0.532	0	1 (9.1)	0.181
Brain	0	0	-	1 (0.5)	0	0.782	0	0	-
Adrenal	1 (0.5)	0	0.771	2 (1.0)	0	0.695	0	0	-
Skin	1 (0.5)	0	0.771	3 (1.5)	0	0.630	0	0	-
Distant lymphatic recurrence	17 (8.5)	1 (5.9)	0.711	16 (8.1)	1 (6.7)	0.841	2 (10.5)	0	0.265

Some patients had more than one recurrence pattern. Bold: Statistically significant.

**Table 4:** Univariate and multivariate analysis of factors affecting OS of all GC patients.

Variables	Univariate Analysis			Multivariate Analysis		
	HR	95% CI	*p* Value	HR	95% CI	*p* Value
Age (year)			**<0.001**			**0.001**
<65	1.00			1.00		
≥65	1.62	1.263–2.074		1.56	1.204–2.030	
Gender			**<0.001**			
Male	1.00					
Female	0.59	0.449–0.784				
Tumor size (cm)			**<0.001**			**0.002**
<5	1.00			1.00		
≥5	2.21	1.705–2.855		1.54	1.172–2.019	
Cell differentiation			0.318			
Poor	1.00					
Moderate	0.85	0.671–1.070				
Well	0.68	0.255–1.856				
Pathological TNM stage			**<0.001**			**<0.001**
I	1.00			1.00		
II	1.38	0.932–2.052		1.17	0.784–1.759	
III	3.61	2.546–5.115		2.94	2.032–4.252	
Adjuvant chemotherapy			1.166			
Yes	1.00					
No	1.17	0.830–1.636				
MSI status			0.944			
MSI/L/S	1.00					
MSI-H	0.99	1.654–1.485				
EBV			0.985			
Negative	1.00					
Positive	0.99	0.555–1.749				

CI: Confidence interval; EBV: Epstein–Barr virus; HR: Hazard ratio; MSI: Microsatellite instability; MSI-H: Microsatellite instability-high; MSI-L/S: Microsatellite instability-low/stable; OS: Overall survival; TNM: Tumor, Node, Metastasis; Bold: Statistically significant.

**Table 5:** Univariate and multivariate analysis of factors affecting DFS of all GC patients.

Variables	Univariate Analysis			Multivariate Analysis		
	HR	95% CI	*p* Value	HR	95% CI	*p* Value
Age (year)			**0.001**			**0.001**
<65	1.00			1.00		
≥65	1.51	1.185–1.925		1.51	1.185–1.925	
Gender			**<0.001**			
Male	1.00					
Female	0.58	0.436–0.759				
Tumor size (cm)			**<0.001**			**0.001**
<5	1.00			1.00		
≥5	2.21	1.713–2.846		1.55	1.187–2.031	
Cell Differentiation			0.271			
Poor	1.00					
Moderate	0.84	0.668–1.060				
Well	0.66	0.243–1.769				
Pathological TNM stage			**<0.001**			**<0.001**
I	1.00			1.00		
II	1.37	0.930–2.016		1.16	0.778–1.716	
III	3.46	2.459–4.861		2.74	1.910–3.936	
Adjuvant Chemotherapy			0.351			
Yes	1.00					
No	1.17	0.841–1.630				
MSI status			0.975			
MSI/L/S	1.00					
MSI-H	1.01	0.668–1.516				
EBV			0.967			
Negative	1.00					
Positive	0.99	0.557–1.752				

CI: Confidence interval; EBV: Epstein–Barr virus; HR: Hazard ratio; MSI: Microsatellite instability; MSI-H: Microsatellite instability-high; MSI-L/S: Microsatellite instability-low/stable; OS: Overall survival; TNM: Tumor, Node, Metastasis; Bold: Statistically significant.

## Abbreviations

AJCC	American Joint Committee on Cancer
CT	computed tomography
CPS	combined positive score
DFS	Disease-free survival
EBER	EBV-encoded small RNAs
EBV	Epstein–Barr virus
GC	Gastric cancer
HP	Helicobacter pylori
IHC	Immunohistochemical
ISH	In situ hybridization
MSI	Microsatellite instability
MSI-H	Microsatellite instability-high
MSS	Microsatellite stable
NGS	Next-generation sequencing
OS	Overall survival
PCR	Polymerase chain reaction
PD-L1	programmed death-ligand 1
TCGA	The Cancer Genome Atlas
TNM	tumor, node, metastasis
UICC	Union for International Cancer Control
